# 2515. Evaluation of the Equivalency of the Oxoid Sulbactam-Durlobactam (10/10µg) Antimicrobial Disc to the CLSI M07 Broth Microdilution Frozen Reference Method

**DOI:** 10.1093/ofid/ofad500.2133

**Published:** 2023-11-27

**Authors:** Katie L Hajek, Amie J Lovatt, Shilpa Bhalerao, Nagina Khan, Dylan T Staats, Megan M Hendrix, Nicole M Holliday, Cindy C Knapp

**Affiliations:** Thermo Fisher Scientific, Oakwood Village, Ohio; Thermo Fisher Scientific, Oakwood Village, Ohio; Thermo Fisher Scientific, Oakwood Village, Ohio; Thermo Fisher Scientific, Oakwood Village, Ohio; Thermo Fisher Scientific, Oakwood Village, Ohio; Thermo Fisher Scientific, Oakwood Village, Ohio; Thermo Fisher Scientific, Oakwood Village, Ohio; Thermo Fisher Scientific, Oakwood Village, Ohio

## Abstract

**Background:**

Sulbactam-durlobactam is an intravenous (IV) investigational drug that is a combination of an IV β-lactam antibiotic (sulbactam), and a novel broad-spectrum IV β-lactamase inhibitor (durlobactam). Sulbactam-durlobactam (SUD) is being developed for the treatment of serious infections caused by *Acinetobacter spp.,* including multidrug-resistant (MDR) strains, and carbapenem-resistant strains. A study was conducted to evaluate the performance and reproducibility of the new Thermo Fisher™ Oxoid™ sulbactam/durlobactam 10/10 μg (SUD20) antimicrobial disc (Thermo Fisher Scientific, Basingstoke, UK) in comparison to the CLSI M07/M100 broth microdilution (BMD) frozen reference method (Thermo Fisher Scientific, Oakwood Village, OH).

**Methods:**

Oxoid SUD20 discs were tested alongside BMD frozen reference panels for the clinical and challenge portions of this study. Two lots of SUD20 discs were utilized to show inter-lot reproducibility as read by three users over three consecutive days of testing. Isolates tested included: 227 *Acinetobacter baumannii*, 5 *Acinetobacter calcoaceticus*, 5 *Acinetobacter pittii*, 7 *Acinetobacter nosocomialis*. Quality control organisms included *A. baumannii* NCTC 13304, and *Escherichia coli* ATCC 25922 using ranges provided in the CLSI M100 document. All isolates were tested in accordance with CLSI M02/ CLSI M07/M100 and EUCAST methodologies.

**Results:**

A categorical agreement of 98% was achieved when comparing the SUD20 disc to the BMD method. Reproducibility results showed 99.3% within-reader and between-reader agreement within 3 mm of the modal value of the combined results. QC results were within the stated limits on each day of testing for each batch, and reader.

**Conclusion:**

The SUD20 disc, when compared to the BMD method, demonstrated an equivalent level of performance against clinical breakpoints as proposed by Entasis Therapeutics. The high categorical agreement obtained by the SUD20 disc indicates that this is an acceptable method for susceptibility testing of sulbactam-durlobactam for isolates of *Acinetobacter spp.*

**Disclosures:**

**All Authors**: No reported disclosures

